# Barriers to Perinatal Palliative Care Consultation

**DOI:** 10.3389/fped.2020.590616

**Published:** 2020-09-22

**Authors:** Franca Benini, Sabrina Congedi, Francesca Rusalen, Maria Elena Cavicchiolo, Paola Lago

**Affiliations:** ^1^Pediatric Pain and Palliative Care Service, Department of Women's and Children's Health, University Hospital Padua, Padua, Italy; ^2^Woman's and Child's Department, Neonatal Intensive Care Unit, University of Padua, Padua, Italy; ^3^Neonatal Intensive Care Unit, Treviso's Hospital, Treviso, Italy

**Keywords:** perinatal palliative care, barriers, newborn, need, neonatal intensive care

## Introduction

Despite that infant mortality rates in western countries have improved over the past decade, 5.8 infant deaths per 1,000 live births are still reported, mainly due to prematurity and congenital anomalies (1, 2). According to recent data collected in the UK, the survival rate of pre-term infants born at 23+0 to 23+6 weeks of gestation and who receive active treatment is 38% (3). According to a registry study of the National Institute of Child Health and Human Development Neonatal Research Network, 20% of infants born at 22–24 weeks of gestation survives without neurodevelopmental impairment (4). Therefore, unfortunately, still a relevant number of newborns admitted to the neonatal intensive care unit (NICU) could have an unfavorable diagnosis and a poor prognosis. This leads to the notion of perinatal palliative care (PnPC) as an emerging field of palliative care. It involves different healthcare providers, including neonatologists, gynecologists, midwives, and nurses, and in a broader view all professionals who provide support strategies to the mother or the family and the fetus/newborn.

PnPC provides supportive care before and after birth and to families when a child is affected by congenital anomalies inconsistent with long-term survival, or when the extreme prematurity or post-natal irreversible disease of the child drastically reduces the possibility of survival (5, 6). Remarkably, in case of life-limiting fetal anomalies, palliative care could start before delivery (7) and up to 85% of parents continue pregnancies with a poor prenatal diagnosis worldwide (8). Therefore, the American College of Obstetricians and Gynecologists and the American Academy of Pediatrics recommend parental informed consent and discussion of all available care options, including PnPC (9).

Despite the importance of PnPC, several barriers to its implementation and utilization exist (10–12). At present, training programs in the field are proposed, the discipline is described as “very important” by hospital leadership but allocation of personnel, space, money, curriculum time remains poor (12).

In this paper, we comment on the barriers that make the access, planning, and development of PnPC services difficult.

## Identification of Barriers and Critical Issues

As a first step, we conducted an evaluation of literature with the aim to identify the most relevant barriers to PnPC and better focus our comment on this topic. In particular, we conducted a literature search on PubMed, TripDatabase, and Google Trend, using different combinations of keywords (e.g., MeSH terms: Perinatal palliative care, Barriers, Newborn, Need, Neonatal Intensive Care, life-limiting illness, end of life, ethical decision; free terms: Gestational age, culture, family, education, model organization, parents, newborn management, information, education, and others). No limitations in the search were applied. The reference lists of the retrieved papers were reviewed to find other papers worth consideration. Authors' own personal collections of literature were also browsed. The identified papers were then selected for inclusion according to their relevance for the topic, as judged by the Authors, in line with the non-systematic and narrative nature of this manuscript.

Based on this literature search and our clinical and organizational experience, we have identified four macro-areas of barriers and critical issues for the development of PnPC, namely: (i) Socio-cultural setting; (ii) Patients and their specific conditions; (iii) Training of healthcare providers (HCP); (iv) Regulatory and political issues.

## Socio-Cultural Setting

### Misperception of the Role of Medicine and the Concept of “Health”

The management of PnPC could be influenced by families', nurses', and physicians' perceptions (8, 13, 14). Indeed, socio-cultural and economic context, and religious and moral values of future parents are taped among the main factors that influence the management of a fetus or an infant eligible to PnPC (13). There are widespread cultural barriers to consider death as an event occurring also in the neonatal/pediatric setting. The child is perceived as a human being at the beginning of his/her life journey and therefore the possible—or certain—fatal event is often neglected by parents and even healthcare providers. Medicine itself is often perceived as having the only aim of “cure,” and the “Healthy” status is only associated with lack of any disease. Conversely, the WHO proposes a wider definition of “Health,” as a state of psychological, physical, and social well-being; Medicine should aim at ensuring this state of health (15). In this perspective, it is important to clarify the notion of the inherent limits of Medicine: in some cases, the adoption of a palliative active care should be preferred over “doing whatever is possible,” i.e., overtreatment (16–18).

The very same definition of “Palliative Medicine” is often misinterpreted as “end-of-life care” by parents of children eligible to PnPC and even by healthcare providers (19). This misinterpretation denies patients the opportunity of appropriate treatment.

### Difficulties in Considering the Baby as a Person Who Can Feel Pain, Distress, Fear, and Suffering

It is widely accepted that, from the second trimester of gestation onwards, the nervous system of the fetus can perceive pain and distress, and these events have a major impact on fetal and post-natal life (20, 21). Therefore, even extremely premature newborns can “feel” the burden of disease, its symptoms, any invasive diagnostic and therapeutic procedure, as well as the distress due to the disease itself and the separation from mother. Unfortunately, the newborn is considered as unable to feel the above situations and therefore the burden associated with disease is neglected or underestimated. Furthermore, by negating newborn's ability to perceive pain and distress, the decision-making process of the clinical strategy becomes impaired. As a consequence, caring for such children requires a multidisciplinary approach that provides information regarding the risk, benefit, and burden for both mother and fetus.

### Poor Information

Information on PnPC and the possibility to “cure” life-limiting or life-threatening diseases is scant, at best, and this lack of information limits the decision-making process by parents of children eligible to PnPC (19).

Moreover, the information that media provide is often only sensationalized news, which can be highly misleading and lacks an honest clinical, ethical and deontological evaluation. This poor information is important both for the patients, their families and for healthcare providers, also since the fear of social judgment limits the possibility of a correct assessment of the patient's status. Furthermore, lack of information and knowledge on PnPC limits the establishment of appropriate interventions by the healthcare system, with the recognition of dedicated units for PnPC.

## Patients and Their Specific Conditions

### Heterogeneity of Clinical Situations and Types of Patients

The PnPC-eligible patients could be divided into three main clinical groups (22): (i) extremely premature at the limits of viability (<23 gestational week); (ii) prenatal/postnatal diagnosis of life-limiting/life-threatening diseases with poor prognosis and/or incompatibility with life; (iii) severe/critical clinical conditions without possibility of improvement, with high complexity of care and needing intensive support for vital functions' maintenance. In the second and third group, a range variety of conditions could be identified: neurological, cardio/pulmonary, and nephrological disorders or other genetic/chromosomal anomalies (23–25). Current medical evidence, experience of the healthcare providers, ethical, and regulatory bodies propose different strategies for the treatment of PnPC-eligible patients, according to their status and the type of life-limiting condition (26). Indeed, the diagnosis of a life-limiting condition can be difficult and can lead to doubts and different hypotheses.

### Communication With Parents and Families

The parents of a child eligible to PnPC, as his/her legal guardians, have the responsibility of taking decisions. However, the inherent level of complexity of PnPC can lead to different views and perceptions between parents and healthcare providers, also due to the substantial variation in prognostic information given by obstetricians and neonatologists (27). This gap is due, at least in part, to the lack of unambiguous sources, tools, and training and it could lead to uncertain diagnostic and prognostic information (28). Several other factors should be taken into account, including the psychological dimension of the issue, the urgency (actual or perceived), the different role, perceptions, and emotions of healthcare providers and parents, the very same definitions of terms, such as “cure,” “disease,” “health,” and “quality of life.” The plethora of different feelings and emotions can be associated with reactions, such as negation of the issue, unrealistic expectations toward medicine, or even delegation of any decision to others and/or impersonal acceptance of the situation. This discrepancy can lead to communication issues between the parents and the healthcare providers, which, in turn, can delay or even deny the possibility of initiating palliative care.

The PnPC team should share the diagnosis, the anticipated prognosis, and potential interventions if any, with compassionate communication that respects patient cultural beliefs and values (29, 30) All the possibilities of care should be presented within a coordinated treatment strategy, focusing on maximizing quality of life, and comfort for the newborn. During counseling, team members should collect the appropriate information in order to give a tailored guidance to the family, free from any personal view and supporting parents in their decision-making process (28, 31). Therefore, team members should ask appropriate questions to explore the family's values, their understanding of the newborn's meaning and quality of life, in order also to establish a relationship based on trust and caring. An advance care plan is shared between parents and PnPC members, a birth plan, and/or a document of family wishes on delivery and resuscitation could be drafted (28).

All care options should be discussed, including pregnancy termination (abortion, within legal limitations), prenatal and postnatal intervention intended to promote survival, and palliative comfort care, which may include interventions to promote comfort and improve quality of life without intending to promote survival (29).

The lack of well-conducted communication can negatively affect the relationship between families of patients and PnPC healthcare providers, and between team members (32).

## Training of Healthcare Providers (HCP)

Dedicated training programs for perinatal palliative care are fundamental to improving the knowledge of healthcare professionals and the overall standard of care delivered to neonates.

In the past years, mounting interest has been given to palliative care, but this has not been paralleled by a growing in training programs; only scant data are available regarding the implementation of the PnPC educational programs.

In 2000, the American Academy of Pediatrics recommended an adequate training in pediatric palliative care and its subspecialties for all pediatricians to enhance the best possible quality of life for children living with a life-threating or terminal condition, using an integrated interdisciplinary approach (33). From 1975 to 2015, 99% of US medical schools offered education in palliative care, but only about 12–15 h of their entire medical school curricula were dedicated to palliative care (34). Similar results were reported for the European setting (35, 36). More data are available regarding training in PnPC for nurses and midwives (37–39), while only scant information regarding educational programs for neonatologist is available (40). According to the above, a structured curriculum dedicated to neonatal–perinatal medicine fellows to develop the complex set of skills required in this setting is eagerly warranted (40).

In a recent review investigating educational programs and events in the field of PnPC (41), the effectiveness of teaching healthcare professionals, methods of evaluating the teaching, and the teaching strategies used were analyzed. In total, 14 studies were included in the analysis, all published between 2002 and 2017. All studies referred as perinatal bereavement education as effective in terms of improvements in health workers of knowledge and communication skills, comfort in providing end-of-life care, and increased perceptions of the emotional needs of bereaved families. However, different evaluation tools were used and hence and it was not possible to determine which program was most effective.

Training programs in PnPC with specific content need to be developed and made available to all professionals who deal with perinatal death and the bereaved families (38, 40). Therefore, a dedicated program in PnPC should be offered to nurses, midwives, and the neonatologist in order to provide the best care for the fetus or infant and his family (42). Many studies reported that the main barriers to the development of pediatric palliative care are the inadequate training in communication and it is likely that this feature is also present in the PnPC field.

## Regulatory and Political Issues

### Organizational Issues

PnPC services represent a new, dynamic, and complex setting of care, which required dedicated time and resources; however, in daily practice, these are not always readily available.

The organization of a PnPC service poses serious challenges and requires the involvement of both local hospitals and the healthcare system. In PnPC, interdisciplinary competent teams are needed, and they should potentially involve different specialists (such as gynecologists, obstetrical specialists, nurses, neonatologists, geneticists, pediatric palliative care specialists, pediatricians, and bioethicists).

Few reports of experiences of well-structured PnPC networking models are available, and only a limited number of these investigated all difficulties and needs. This complex networking of different experiences needs the definition of shared protocol, dedicated organizational models, and adequate training. Meetings should begin in the antenatal phase and continue later, in order to identify and pursue a mutually agreed and appropriate care program. Dedicated time and appropriate places are needed for multidisciplinary meetings, and they may also help ensure a good communication between families of patients and team members, during the entire course of the patient's disease.

A quiet place should be available for parents during the infant dying process (6). Hospice concepts should be applied to neonatal care, providing a private family room where other family members can gather, and religious support can be given (43).

### Resources Issues

From their moment of establishment, neonatology services have been characterized by continuous medical innovations, in a fast-growing field. However, only scant resources are available for this setting, requiring proper allocation (44). Moreover, literature offers complex networking models for PnPC services (45), making it difficult to estimate the costs for the public healthcare system, as well as the different social costs.

### (Lack of) Regulations

The lack of dedicated regulations, which clearly define the right of children to PnPC, makes them, in many counties, an optional opportunity and not a duty of the healthcare system. Therefore, access to PnPC is largely dependent on single institutions, which further limits the diffusion of PnPC.

## Conclusions

In this brief analysis, we identified and commented on many of the barriers currently slowing the development of PnPC programs. This issue is challenging, also due to the high heterogeneity of PnPC; however, we can propose some actions that may help in establishing proper PnPC programs.

- Increase the recognition of the actual lack of PnPC programs, in order to increase awareness of this setting.
- Recognize the inherent complexity of PnPC, making it necessary to establish specific training and services and collecting dedicated resources. Improvisation is not feasible in the setting of PnPC.
- Increase social awareness on PnPC, which aims at overcoming prejudices and providing the tools for proper reflections and decision-making.
- Provide proper training on PnPC, in terms of clinical, ethical, organizational, and communicational skills in order to make them able to pursue the best interest of the newborn with life-limiting/life-threatening disease.
- Promote research in PnPC and the collection of shared data, in order to find new tools applicable in different centers and settings.

## Author Contributions

FB: study design, data analysis, data interpretation, and writing. SC: literature search, data analysis, data collection, and writing. FR: literature search, data collection, and writing. MC: literature search, data interpretation, and writing. PL: literature search, study design, data interpretation, and writing. All authors: approved the final version of the draft.

## Conflict of Interest

The authors declare that the research was conducted in the absence of any commercial or financial relationships that could be construed as a potential conflict of interest.
